# Exosomal miRNA Signatures for Late-Onset Acute Graft-Versus-Host Disease in Allogenic Hematopoietic Stem Cell Transplantation

**DOI:** 10.3390/ijms19092493

**Published:** 2018-08-23

**Authors:** Seiichiro Yoshizawa, Tomohiro Umezu, Yuu Saitoh, Moritaka Gotoh, Daigo Akahane, Chiaki Kobayashi, Junko H. Ohyashiki, Kazuma Ohyashiki

**Affiliations:** 1 Department of Hematology, Tokyo Medical University, Tokyo 160-0023, Japan; t_umezu@tokyo-med.ac.jp (T.U.); yusaitoh@tokyo-med.ac.jp (Y.S.); gotohm@wj8.so-net.ne.jp (M.G.); daigoakahane@msn.com (D.A.); chiaki-k@tokyo-med.ac.jp (C.K.); ohyashik@rr.iij4u.or.jp (K.O.); 2 Department of Advanced Cellular Therapy, Tokyo Medical University, Tokyo 160-0023, Japan; junko@hh.iij4u.or.jp

**Keywords:** late-onset acute graft-versus-host disease, exosomal miRNA, hematopoietic stem cell transplantation

## Abstract

Recent studies have demonstrated that exosomal microRNAs (miRNAs) have the potential of facilitating molecular diagnosis. Currently, little is known about the underlying mechanism behind late-onset acute graft-versus-host disease (LA GVHD). Identifying differentially expressed miRNAs in exosomes should be useful for understanding the role of miRNAs in this disease. This study was established to investigate the relevance of miRNAs in exosomes derived from patients developing LA GVHD after allogeneic hematopoietic stem cell transplantation (HSCT). Plasma samples were collected from patients with LA GVHD (*n* = 5), non-GVHD (*n* = 5), and controls (*n* = 8) for exosomal miRNA expression profiling using a TaqMan low-density array; the results were validated by quantitative reverse transcription polymerase chain reaction (RT-PCR). We analyzed exosomal miRNAs differentially expressed among these three groups. MirTarBase was employed to predict potential target genes of the miRNAs specific for LA GVHD. We detected 55 miRNAs that were differentially expressed with a significant change >2.0-fold between LA GVHD and non-GVHD. Of these, we selected the 10 miRNAs (miR-423-5p, miR-19a, miR-142-3p, miR-128, miR-193b, miR-30c, miR-193a, miR-191, miR-125b, and miR-574-3p) with the most significant differential expression. Using quantitative RT-PCR, we further identified that miR-128 was significantly upregulated at the onset of LA GVHD compared with that in normal controls and is a promising diagnostic marker of LA GVHD, with an area under the curve (AUC) value of 0.975. MirTarBase analysis revealed that the predicted target genes of miR-128 are involved in the immune system and inflammation. Increased expression of miR-128 may serve as a novel, noninvasive biomarker for early LA GVHD diagnosis.

## 1. Introduction

The occurrence of severe graft-versus-host disease (GVHD) after allogeneic hematopoietic stem cell transplantation (HSCT) leads to a poor prognosis. Recently, with this expansion in the utilization of allogeneic HSCT, such as the introduction of reduced-intensity conditioning and donor lymphocyte infusions, late-onset acute and chronic GVHD has been reclassified based on the exact clinical manifestation instead of the time of onset [1]. According to the new classification using National Institutes of Health (NIH) consensus criteria established in 2005, patients with de novo acute GVHD occurring beyond day 100 post-transplantation are considered to have late-onset acute GVHD (LA GVHD) [1]. In accordance with the NIH definition, 20–40% of chronic GVHD cases are reconsidered to be LA GVHD. A few reports have shown that LA GVHD is also associated with greater nonrelapse mortality after allogeneic HSCT than non-GVHD [2,3]. However, there is still an insufficient understanding of the association between LA GVHD and a lower incidence of relapse caused by the induction of a beneficial graft-versus-tumor (GVT) effect. To improve long-term survival, intensive efforts have been made to prevent GVHD without losing the GVT effect. Thus, several molecular biomarkers for diagnosis and prediction of the severity and prognosis of GVHD have been explored [4,5,6,7].

MicroRNAs (miRNAs) are small noncoding RNA molecules that regulate post-transcriptional gene expression by degrading or suppressing their target mRNA [8]. Recently, it has been widely recognized that some circulating miRNAs are included in the content of endosome-derived extracellular vesicles including exosomes. Exosomes are secreted by many types of cells into various biological fluids, such as serum and plasma [9,10]. The demonstration that exosomes contain not only proteins, RNAs, and lipids, but also functional miRNAs indicated that miRNAs could participate in intercellular communication and elicit immune responses by their transport between cells via exosomes [11,12]. Therefore, analysis of the alterations of exosomal miRNAs in the context of various diseases has the potential to facilitate molecular diagnosis [13,14].

In recent studies, miRNA profiling identified a GVHD-specific miRNA signature that can control the regulation of classic acute GVHD in allogeneic immunity. In particular, miR-155, known as an immune-related microRNA, and miR-15a/16 and miR-17-92a were shown to be closely involved in the pathophysiology behind the development of classic acute GVHD in a xenogeneic GVHD murine model [15,16,17]. Moreover, recent studies have demonstrated that specific plasma miRNA signatures, such as miR-423, miR-199a-3p, miR-93, miR-377, and miR-586, could serve as independent biomarkers for the prediction, diagnosis, and prognosis of hematologic diseases and classic acute GVHD [18,19]. Despite the fact that the roles of miRNAs in classic acute GVHD after allogeneic HSCT have been extensively studied over the last few years, the relevance and expression profile of miRNAs in LA GVHD have not yet been elucidated. Because a number of previous studies investigated the role of miRNAs as potential predictive biomarkers in acute GVHD, we aimed to determine the clinically relevant exosomal miRNA profile in patients developing LA GVHD after allogeneic HSCT.

## 2. Results

### 2.1. Patient Characteristics

A summary of patients’ clinical characteristics is presented in Table 1. The median age of all patients was 58.5 years (range, 26–70 years) and the median onset of LA GVHD was 165 days (range, 117–238 days) after allogeneic HSCT. GVHD prophylaxis included mainly calcineurin inhibitor with or without short-term methotrexate. Overall, four of the five of patients with LA GVHD developed severe GVHD (grades III–IV) and no patients achieved remission despite second-line therapy. Four patients died from GVHD deterioration, while the remaining one patient died from post-transplantation lymphoproliferative disorder.

### 2.2. miRNA Expression Profiling at LA GVHD Diagnosis

**Figure 1:**
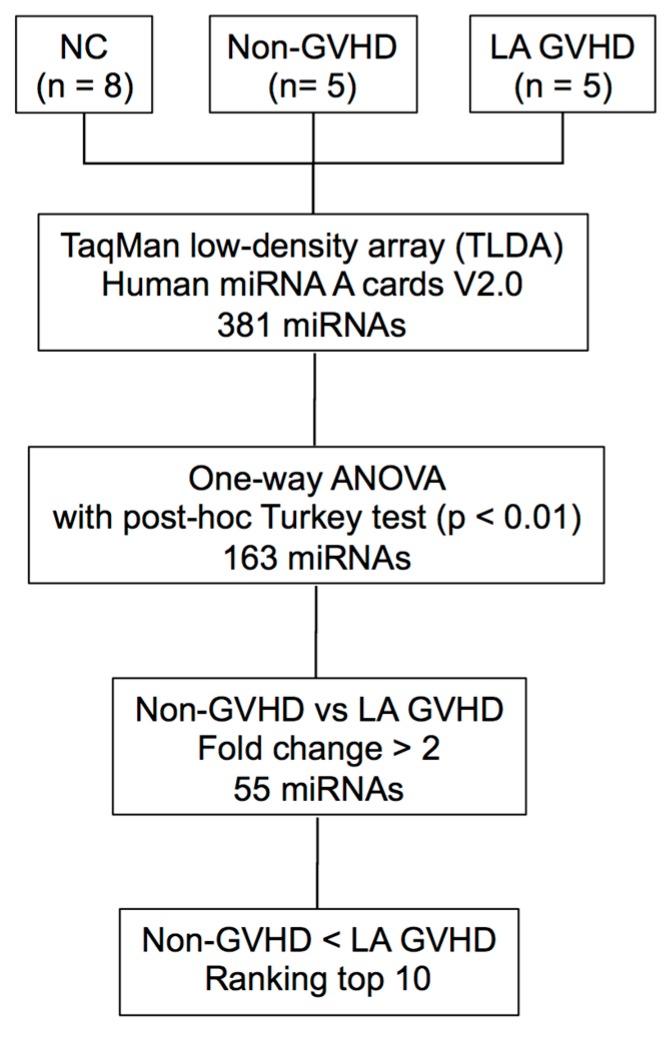
Flow diagram for the identification of late-onset acute GVHD-specific miRNAs. The exosomes fractions were isolated from 18 plasma samples of HSCT patients and healthy volunteers, and the expression profile of 381 exosomal miRNAs were analyzed. To validate candidate miRNAs, we performed TLDA and further narrowed down the miRNAs to those significantly differentially expressed by at least two-fold.

**Figure 2:**
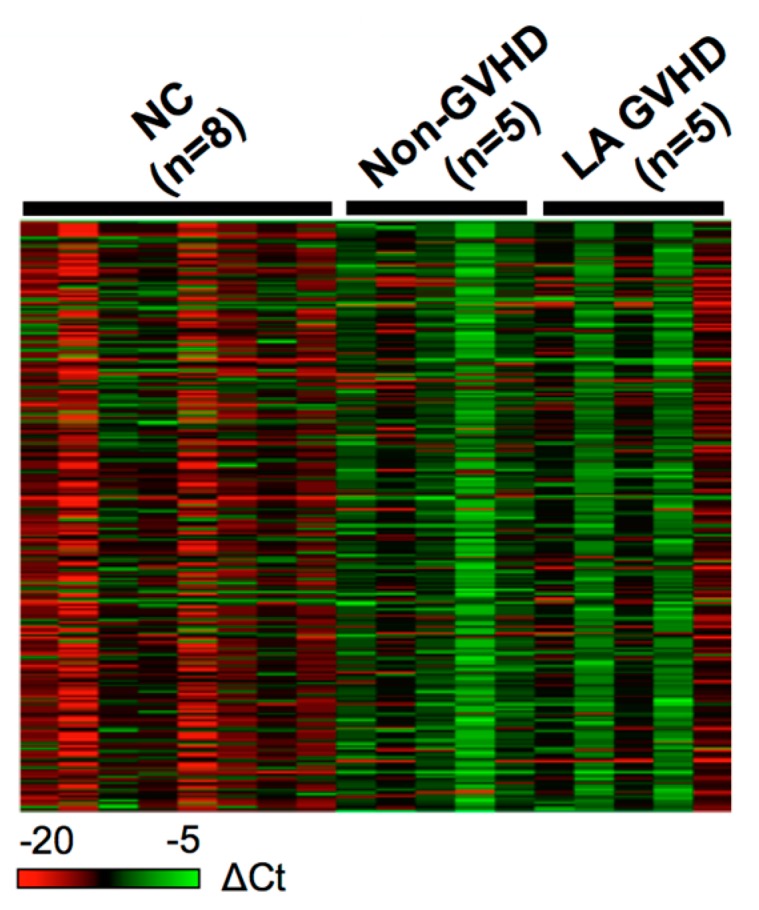
miRNA expression profiling using TaqMan miRNA Array (Thermo Fisher Science, Waltham, MA, USA). Exosome fraction taken from plasma samples of 10 patients who underwent allogeneic HSCT (5 late-onset acute GVHD and 5 non-GVHD), with 8 normal controls being included. A differential expression pattern was found between late-onset acute GVHD patients and non-GVHD patients. Using SDS (version 2.4) and DataAssist software (version 3.01, Thermo Fisher, version, Waltham, MA, USA), we calculated the expression of miRNAs based on their Ct value normalized by the Ct value of ath-miR-159. Data were analyzed with GeneSpring software.

The procedure for selecting candidate miRNAs is illustrated in Figure 1. To identify candidate miRNAs with altered expression in LA GVHD patients, miRNA expression profiling was performed on exosome fractions taken from plasma samples of 10 allogeneic HSCT patients (5 LA GVHD and 5 non-GVHD) and 8 normal subjects. We then compared exosomal miRNA expression in LA GVHD patients with that in non-GVHD patients using a TaqMan miRNA Array (NCBI, Gene Expression Omnibus (GEO); GSE110435). The heat map in Figure 2 demonstrates that the miRNA signature in LA GVHD group tended to show high expression. Among these three groups, a total of 163 differentially expressed miRNAs were identified using GeneSpring software. We confined our analysis to miRNAs for which the expression level exhibited a fold change (FC) of >2.0 in exosomes of the LA GVHD group compared with that in the non-GVHD group. Interestingly, for all of the 55 selected miRNAs, the expression was upregulated in the LA GVHD group. We then evaluated the 10 miRNAs with the highest FC (miR-423-5p, miR-19a, miR-142-3p, miR-128, miR-193b, miR-30c, miR-193a, miR-191, miR-125b, and miR-574-3p), as shown in Table 2. Of these miRNAs, miR-423-5p, miR-19a, and miR-142, which were most strikingly differentially expressed, were previously reported to play important roles in acute GVHD [18,20,21].

### 2.3. Comparison of Exosomal miRNAs Differentially Expressed among LA GVHD, Non-GVHD, and Healthy Volunteers

**Figure 3:**
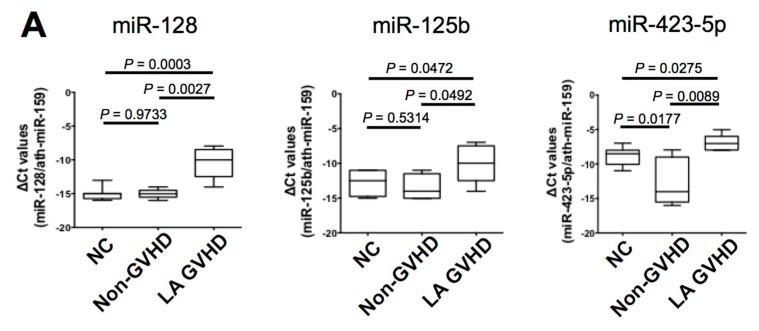

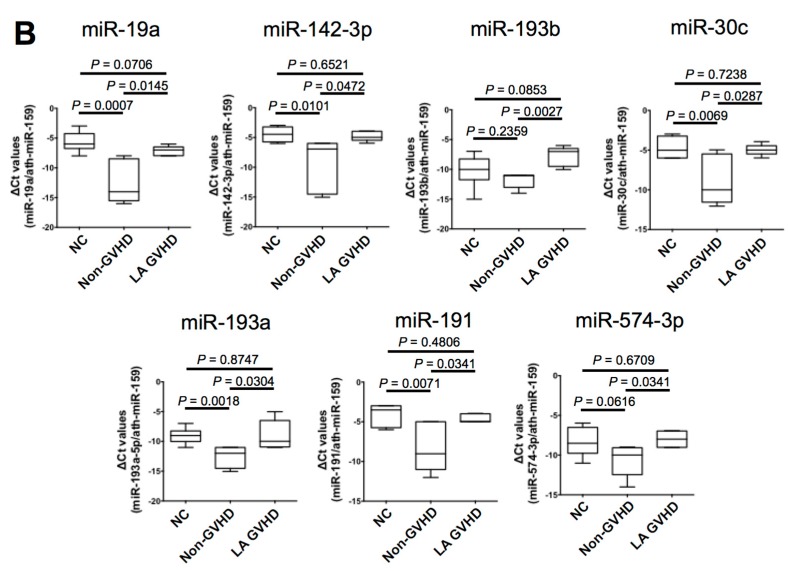
This microRNA expression according to late-onset acute GVHD (LA GVHD) vs. non-GVHD vs. normal controls at the onset of late-onset acute GVHD. Box plots depict the 75th percentile, median, and 25th percentile values; and whiskers represent minimum to maximum expression. *p* values were calculated using the independent two-sample *t*-test. (**A**) The expression levels of three miRNAs (miR-128, miR-125b, and miR-423-5p) were significantly higher in the exosomes of LA GVHD than in normal controls. The expression level of miR-423-5p showed downregulation in the non-GVHD groups when compared with normal controls. Accordingly, two miRNAs (miR-128, miR-125b) were chosen as candidate miRNAs specific for LA GVHD. (**B**) No significant differences of expression of seven miRNAs (miR-19a, miR-142-3p, miR-193b, miR-30c, miR-193a, miR-191, and miR-574-3p) were observed between LA GVHD and normal controls.

To validate the findings on the LA GVHD-specific exosomal miRNAs, we assessed the 10 exosomal miRNAs that were most highly differentially expressed among the three groups. We adopted the screening method for selecting LA GVHD-specific exosomal miRNAs as follows: (1) significant difference in the expression of miRNAs is observed between LA GVHD and healthy volunteers (normal control); or (2), no significant difference in the expression of miRNAs is observed between the non-GVHD and healthy volunteers (normal control). We firstly compared samples obtained from patients at the diagnosis of LA GVHD with those from healthy volunteers (normal control) and found that three miRNAs (miR-423-5p, miR-128, and miR-125b) were significantly overexpressed in the exosomes of those with LA GVHD (*p* < 0.05) when compared with the levels in healthy subjects (Figure 3A). Meanwhile, none of the remaining seven miRNAs showed significant differences in expression between LA GVHD and healthy volunteers (Figure 3B). Subsequently, we compared samples obtained from non-GVHD patients with those from healthy volunteers in Figure 3A. No significant difference in the expression of miR-128 and miR-125b was observed between the non-GVHD and healthy volunteers, while miR-423-5p showed downregulation in the non-GVHD group when compared with healthy volunteers. Therefore, two miRNAs, miR-128 and miR-125b, were chosen as candidate miRNAs specific for LA GVHD; For further analysis, we focused on the clinical implications of these two dysregulated miRNAs in LA GVHD.

### 2.4. Quantification of Individual miRNA by Real-Time Quantitive RT-PCR

**Figure 4:**
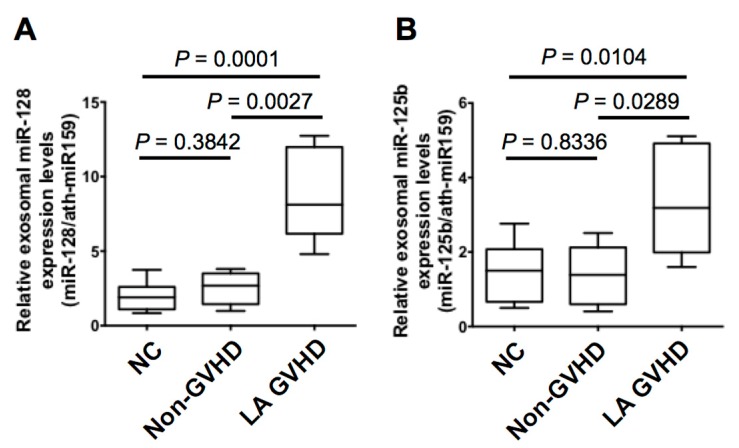
(**A**) The expression level of miR-128 was significantly upregulated in the exosomes of late-onset acute GVHD when compared with that in non-GVHD (*p* = 0.0027) and in healthy subjects (*p* < 0.005). (**B**) The expression level of mir-125b was significantly upregulated in the exosomes of late-onset acute GVHD when compared with that in non-GVHD (*p* = 0.0289) and in healthy subjects (*p* = 0.0104).

**Figure 5:**
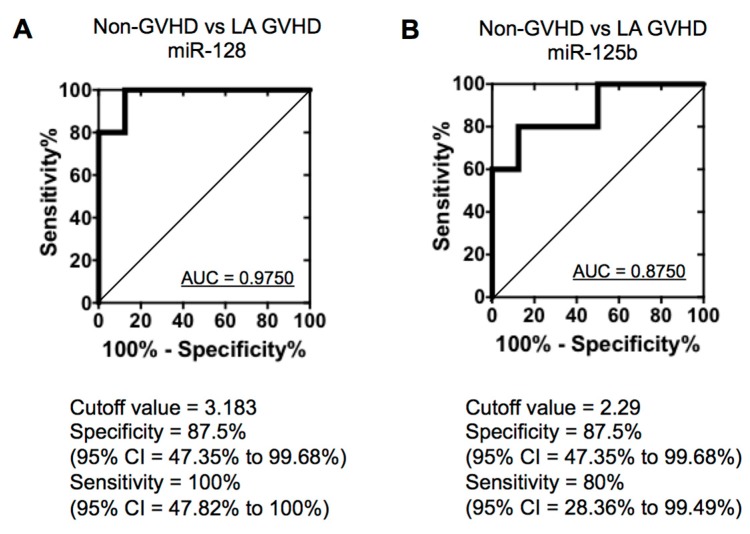
Association between exosomal miRNA and late-onset acute GVHD. (**A**) ROC curve for the identification of patients with late-onset acute GVHD versus non-GVHD using miR-128. (**B**) ROC curve for the identification of patients with late-onset acute GVHD versus non-GVHD using miR-125b.

To confirm the specific deregulated miRNAs change obtained from the TaqMan low-density array (TLDA), we quantified miR-128 and miR-125b expression by quantitative real-time PCR (qRT-PCR). There were no significant differences in miR128 (Figure 4A) and miR-125b (Figure 4B) expression between the normal control and non-GVHD group (*p* = 0.3842 and 0.8336, respectively). In contrast, miR-128 and miR-125b were significantly increased in LA GVHD patients (LA GVHD vs. non-GVHD, *p* = 0.0027 and 0.0289; LA GVHD vs. NC, *p* = 0.0001 and 0.0104, respectively) (Figure 4A,B). To estimate cut-off values for the two miRNAs (miR-128, miR-125b) in exosomes for distinguishing between LA GVHD and non-GVHD groups, we performed receiver-operating characteristic (ROC) curve analysis. The ROC curve for miR-128 showed extremely high diagnostic accuracy, with an area under the ROC curve of 0.975 and diagnostic sensitivity of 100% [95% confidence interval (CI), 47.82–100%] and specificity of 87.5% (95% CI, 47.35–99.68%) (Figure 5A). Similarly, the ROC curve for miR-125b showed an AUC of 0.975 and diagnostic sensitivity and specificity of 80% (95% CI, 28.36–99.49%) and 87.5% (95% CI, 47.35–99.68%), respectively (Figure 5B). The cut-off values were thus determined to be 3.183 for miR-128 and 2.29 for miR-125b, which were used in the following analysis. Our data suggest that altered expression of miRNAs may serve as a diagnostic tool for LA GVHD in HSCT practice.

### 2.5. Sequential Analysis of Exosomal miRNAs Post-Allogenic HSCT

**Figure 6:**
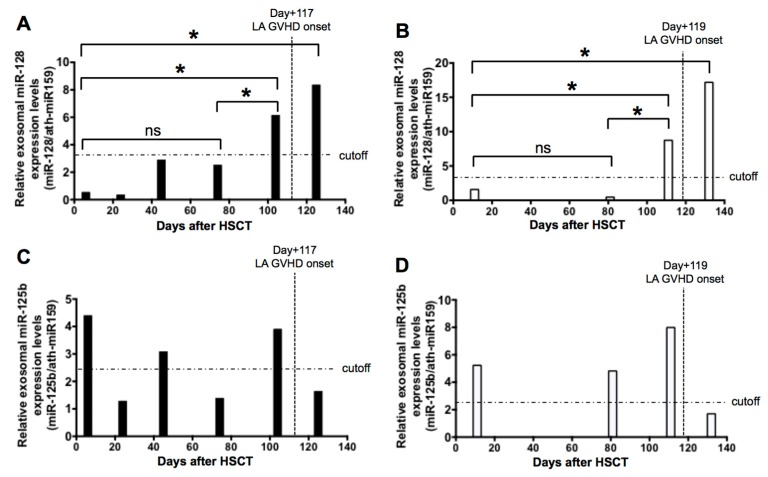
Bar graphs show relative expression levels of each miRNA at each time point after HSCT. (**A**,**B**) The dynamic changes in exosomal miR-128 prior to the late-onset acute GVHD diagnosis. The information of PN 3 was day +7, day +25, day +46, day +70, day +105, and onset. The level of miR-128 was upregulated over the cut-off level at day +105 (**A**). The information of PN 4 was day +11, day +81, day +111, and onset. The level of miR-128 was upregulated over the cut-off level at day +111 (**B**). (**C**,**D**) The changes of exosomal miR-125b varied widely after HSCT. * *p* < 0.005, ns: Not significant.

To further explore whether exosomal miRNAs can be used as predictive biomarkers of LA GVHD, we next measured miR-125b and miR128 expressions sequentially in exosomes of available serum samples taken from two transplanted patients (patient number PN 3 and PN 4 in Table 1). As illustrated in Figure 6, we observed that the level of miR-128 was significantly upregulated approximately a week or two weeks before the onset of LA GVHD in PN 3 and PN 4 (Figure 6A,B). When we used the cut-off value (3.183) obtained from the ROC analysis, the levels of miR-128 expression on days +25, +46, and +70 of PN 3 were under this value but exceeded the cut-off value at day +105 in the prediagnosis of LA GVHD (Figure 6A). In addition, the levels of miR-128 expression on days +11 and +81 of PN 4 were under that value but exceeded the cut-off value at day +111 in the prediagnosis of LA GVHD (Figure 6B). On the other hand, the miR-125b expression level was upregulated immediately after HSCT, and elevated miR-125b expression was unable to predict LA GVHD (Figure 6C,D). This suggests that increased miR-128 expression in patients after HSCT may be a predictive marker prior to the onset of LA GVHD. Finally, the validity of miR-128 as a biomarker was confirmed by using it to discriminate LA GVHD patients from non-GVHD cases, although we should consider the bias of stem cell source.

### 2.6. Prediction of the Target Genes of Candidate miRNAs

This study indicated that upregulated miR-128 is crucial as an LA GVHD-specific miRNA. The clinical relevance of miRNAs involved in the development of LA GVHD was not evident. Thus, MirTarBase was used to find predicted target genes of miR-128 in order to determine the biological significance of this miRNA. More than 100 target genes were extracted by MirTarBase, and the target genes for which strong evidence existed in the results of the search are summarized in Table 3. Accordingly, 12 genes, including B-cell specific Moloney murine leukemia virus integration region 1 (BMI1), F-box, and tryptophan-aspartic acid (WD) repeat domain containing 7 (FBXW7), were experimentally validated by a luciferase reporter assay in the literature [22,23,24,25,26,27].

## 3. Discussion

Recently, the definition of GVHD was revised in the NIH consensus criteria, with the new category of “late-onset” acute (LA) GVHD being established. Given the short history of this category, the pathophysiology of LA GVHD is still poorly understood. Although the diagnosis of GVHD is judged based on clinical manifestations and pathological findings of the tissue sections from the intestines or liver, it is difficult to provide an accurate pathological diagnosis of GVHD. For this reason, pathological findings have greatly affected various factors of radiation damage, drug injury, and viral infection, presenting a diagnostic dilemma. Therefore, there is an urgent need of exploring reliable, noninvasive biomarkers of GVHD. In the context of GVHD after allogeneic HSCT, biomarker research has progressed through a variety of techniques, such as immunocompetent cells (CD4 helper T cells, regulatory T cells), plasma (soluble IL-2R), and inflammatory cytokines (IFN-γ, TNF-α). For example, Paczesny et al. identified that a panel of four biomarkers (IL-2Rα, TNFR1, IL-8, HGF) in serum was useful for the diagnosis of acute GVHD [4]. This group also found that the suppression of tumorigenicity 2 (ST2) could distinguish treatment-resistant GVHD patients, and that ST2 levels were associated with nonrelapse mortality [6]. Moreover, a specific plasma miRNA signature for classic acute GVHD was recently determined by Xiao et al. [18]. To date, the identification of altered miRNAs has been thought to contribute to the HSCT-related pathogenesis of acute GVHD; however, the mechanism underlying LA GVHD remains to be elucidated. The above-mentioned results provided us with a potential tool as a new liquid biopsy technique.

Because a number of previous studies investigated the role of exosomes and exosomal miRNAs as potential predictive biomarkers in acute GVHD [20,28], we aimed to elucidate the possible role of miRNAs specifically in LA GVHD patients. Exosomes containing miRNAs reflect the behavior of certain types of cells, so a survey of changes of miRNAs in exosomes should promote our understanding of clinical conditions. In this context, technical advances in exosome separation and characterization have also recently been achieved, enabling exosomal miRNAs to be easily detected by high-throughput techniques [29]. Additionally, Kordelas et al. reported an attractive treatment using mesenchymal stem cell (MSC)-derived exosomes for therapy-refractory GVHD [30]. We also previously reported that many miRNAs have been identified as noninvasive, diagnostic, prognostic, and predictive markers for hematologic malignancy [31,32,33]. In the current study, we explored the LA GVHD-specific exosomal miRNA signature for LA GVHD compared with that in non-GVHD. We also performed bioinformatic analysis to investigate the potential molecular mechanisms behind LA GVHD and identify molecular target genes. Our results of validation by qRT-PCR showed that miRNA-128 significantly increased in LA GVHD patients. The miRNA miR-128 is well known as a brain-enriched miRNA, which plays a crucial role in the development of the nervous system [34]. The miRNA miR-128 has also been previously demonstrated to be dysregulated in tissues and blood samples in several malignant tumor patients and to be correlated with tumor progression [35,36,37]. In the current study, it was impossible to conduct a functional analysis of miR-128. Hence, using MirTarBase, we instead searched for potential target genes to investigate the mechanism of miR-128 (Table 3).

We perfomed ROC analysis to assess the diagnostic capacity of exosomal miRNAs. Exosomal miR-128 exhibited its potential in discriminating LA GVHD from non-GVHD groups. When the expression level of candidate miRNAs was analyzed sequentially in a subset of LA GVHD patients during allogeneic HSCT, miR-128 was shown to increase over the cut-off value earlier than the development of the disease. However, our results must be interpreted with caution, because this study was limited by the relatively small number of samples from LA GVHD patients and absence of a validation cohort of any sort. After all, molecular mechanisms on the dysregulation of exosomal miRNAs in patients with LA GVHD could not be fully provided. Also, candidate miRNAs were then examined longitudinally in only two patients over time. Thus, these results need to be further studied with large cohort.

The results of the top 10 miRNAs obtained by TLDA included several characterized miRNAs (miR-423-5p, miR-19a, and miR-142) known to be dysregulated in acute GVHD [18,20,21]. In particular, miR-423-5p, which is involved in the immune response of acute GVHD incidence, was elevated up to 49-fold in exosomes of LA GVHD patients in our study. Crossland et al. reported differential expression of miR-423 in exosomes at day 14, after HSCT had shown potential as a predictive biomarker for the occurrence of acute GVHD [20]. Therefore, the pathology of LA GVHD may resemble that of acute GVHD caused by donor T-cell alloreactivity in light of miRNAs. However, the biology of GVHD is complex. In other words, miRNAs also regulate various molecular targets, including normal development, differentiation, and maturation of hematopoietic cells in the immune system [38]. The difference of miRNA expression may influence the process of immune reconstitution after allogeneic HSCT. Moreover, the changes of immune-related miRNAs may be due to the dosage of immunosuppression. The dysregulated miRNAs when LA GVHD occurs may be repressed by immunosuppressive regimen. However, it is difficult to evaluate whether the level of this miRNA returns to baseline upon therapeutic intervention because of the uncontrollable nature of LA GVHD. It is a challenge for future study to clarify the association between miRNA expression and response to therapy. Although it is difficult to define miRNAs specific to LA GVHD in very complex medical cases, and in the small number of transplanted patients in the current study, exosomal miR-128 may be related to the immunoregulation of LA GVHD.

In summary, this study demonstrated that the levels of several exosomal miRNAs change at the time of LA GVHD onset after HSCT. Although a longitudinal series of blood samples was taken from 2 patients only, upregulation of miR-128 expression level preceded of LA GVHD incidence. In particular, our results suggest that biological alterations of miR-128 in exosomes may serve as potent predictive biomarkers for close monitoring of the onset of LA GVHD. We consider that this work could provide a foundation for revealing the importance of miRNA in the pathophysiology of LA GVHD. GVHD remains a major cause of transplant-associated complications, which can lead not only to lethal organ damage, but also to a decreased quality of life. Inhibition of certain exosomal miRNAs, such as miR-128, might be considered as a novel potential treatment of LA GVHD to target the immune system and inflammatory signaling, to control GVHD. However, further studies are needed to elucidate the molecular mechanisms behind the development of LA GVHD.

## 4. Materials and Methods

### 4.1. Ethics

The use of patient samples was approved by the Institutional Review Board of Tokyo Medical University (IRB no. 1979, approved on 28 March 2011). Written informed consent was obtained from all of the participants before the collection of specimens, in accordance with the Declaration of Helsinki.

### 4.2. Diagnosis of Late-Onset Acute GVHD

The diagnosis of LA GVHD here was based on clinical symptoms or was histologically proven by biopsy in the target organs, as described previously [1]. Patients with typical manifestations of classic acute GVHD occurring beyond 100 days after transplantation and persistent, recurrent acute GVHD were considered as having LA GVHD, in accordance with the new classification based on NIH consensus criteria [1].

### 4.3. Isolation of Exosome Fractions from Plasma Sample of Patients

Ten patients with hematological malignancies who underwent allogeneic HSCT between February 2012 and November 2013 were included in this study, which consisted of five patients with LA GVHD (gut, *n* = 2; liver, *n* = 2; skin and gut, *n* = 1) and five stable patients without GVHD symptoms (non-GVHD) under immunosuppression. All samples were collected sequentially after HSCT. For patients with LA GVHD, we evaluated blood samples obtained at the onset of this disease. No treatment for LA GVHD had been given at the time of sampling. For comparison, blood samples from HSCT patients without GVHD symptoms and other complications were used. In addition, eight healthy controls were also randomly selected in our hospital. We extracted exosome fractions from 200 µL of plasma using Total Exosome Isolation Reagent (Invitrogen, Carlsbad, CA, USA) and then assessed exosomal miRNA expression in a subset of samples, as described previously [11].

### 4.4. Exosomal miRNA Profiling

Total RNA was isolated from exosomes fractions of five patients with LA GVHD and five patients without GVHD (non-GVHD) using the miRNeasy Mini kit (Qiagen, Hilden, Germany). Exosomes were diluted with 700 μL of QiaZol. After 5 min of incubation, 1 μL of 1 nM ath-miR-159 (Hokkaido System Science, Hokkaido, Japan) was added to the aliquot followed by vortexing for 30 s and incubation for 5 min. Subsequent phenol extraction and centrifuge filtration were performed, in accordance with the manufacturer’s instructions (Qiagen, Hilden, Germany). The RT reaction and pre-amplification step were set up in accordance with the manufacturer’s instructions (Thermo Fisher Science, Waltham, MA, USA). miRNAs were reverse-transcribed with Megaplex Prime Pools (Human Pools A v2:1; Thermo Fisher Sciences, Waltham, MA, USA).

### 4.5. TaqMan Loq-Density Array Screening

The miRNA expression profiles were determined with a TaqMan miRNA Array Human Card A (Thermo Fisher Sciences, Waltham, MA, USA). Quantitative RT-PCR was performed on an Applied Biosystem 7900 HT thermocycler, in accordance with the manufacturer’s recommended program [31]. Using SDS2.2 software and Data Assist (version 3.01, Thermo Fisher Sciences, Waltham, MA, USA), the expression of exosomal miRNAs was calculated based on cycle threshold (Ct) values normalized by those of ath-miR-159, which was spiked in each exosomal sample. Data analysis was performed using GeneSpring^®^ software (Version 12.1, Agilent Technologies, Palo Alto, CA, USA) and R software (https://www.r-project.org, accessed on 27 Arpil 2017). The Benjamini–Hochberg algorithm was used for the estimation of false discovery rates, as we reported previously [31].

### 4.6. Real-Time Quantitative RT-PCR for Candidate miRNAs

We determined the levels of the individual miRNAs by real-time quantitative RT-PCR with a TaqMan MicroRNA Assay (Thermo Fisher Sciences, Waltham, MA, USA) and the following miRNA-specific stem-loop primers: hsa-miR-128 (assay ID: 002216, Ambion, Austin, TX, USA) and hsa-miR-125b (assay ID: 000449, Ambion, Austin, TX, USA). Subsequently, quantitative real-time PCR was performed with an ABI Prism 7900 sequence detection system (Thermo Fisher Sciences, Waltham, MA, USA). The reaction was initiated by incubation at 95 °C for 2 min, followed by 50 cycles of 95 °C for 15 s and then 60 °C for 1 min. All reactions were run in duplicate. Mean (Ct) values for all miRNAs were quantified with sequence detection system software (SDS version 1.02; Thermo Fisher Sciences, Waltham, MA, USA) [31]. The expression of all miRNAs was normalized to miR-159, which was stably detected in all samples, yielding a −ΔΔCt value that was calculated by subtracting the −ΔΔCt value of the normal samples from the respective −ΔCt values of patient samples. The expression of all miRNAs was normalized using the 2^−ΔΔCt^ method.

Following the identification of differentially expressed miRNAs, the predicted target genes of these altered miRNAs were investigated using the experimentally validated miRNA–target interactions database MirTarBase (http://mrtarbase.mbc.nctu.edu.tw/, accessed on 11 September 2017).

### 4.7. Statistical Analysis

Data are expressed as mean ± standard deviation (SD). Multiple groups were compared by one-way analysis of variance (ANOVA) with post hoc Tukey’s test. Statistical analysis was performed using GeneSpring software, and miRNAs with both a ΔCt of either >1.0 or <−1.0 and a P value of less than 0.05 were deemed to be differentially expressed. The area under the curve (AUC) of the receiver-operating characteristic (ROC) curve was calculated. GraphPad Prism software (version 5c for Macintosh; GraphPad Software Inc., La Jolla, CA, USA) was used for statistical analysis.

## Figures and Tables

**Table 1:** Characteristics of hematopoietic stem cell transplantation (HSCT) patients.

	Diagnosis	Age (y)	Gender	Donor	HLA Sero Compatibility	Stem Cell Source	Conditioning	GVHD Prophylaxis	Days of LA GVHD Onset	Days of Sampling	Target Organ	Grade
**Late-Onset Acute GVHD Group**												
PN 1	AML	33	M	Matched unrelated	6/6	BM	Myeloablative	CsA + st-MTX	+238	+248	Liver	IV
PN 2	NHL	38	M	Matched related	6/6	PBSC	Reduced intensity	CsA + st-MTX	+180	+221	Liver	IV
PN 3	AML	57	M	Mismatched	5/6	BM	Myeloablative	Tac + st-MTX	+117	+136	Gut	III
PN 4	ALL	56	F	Mismatched	4/6	Umbilical cord blood	Myeloablative	Tac	+119	+132	Skin + gut	IV
PN 5	ALL	60	F	Mismatched	4/6	Umbilical cord blood	Myeloablative	Tac	+165	+174	gut	II
**Non-GVHD Group**												
PN 6	ALL	62	M	Matched unrelated	6/6	BM	Reduced intensity	CsA + st-MTX		+42		
PN 7	ALL	26	F	Matched unrelated	6/6	BM	Myeloablative	CsA + st-MTX		+41		
PN 8	AML	60	M	Mismatched	4/6	Umbilical cord blood	Myeloablative	Tac		+49		
PN 9	AML	62	M	Mismatched	5/6	PBSC	Reduced intensity	Tac + st-MTX		+41		
PN 10	AML	70	F	Mismatched	4/6	Umbilical cord blood	Reduced intensity	Tac		+45		

AML, acute myeloid leukemia; NHL, non-Hodgkin lymphoma; ALL, acute lymphoblastic leukemia; BM, bone marrow; PBSC, peripheral blood stem cell; CsA, cyclosporine A; Tac, tacrolimus; MTX, methotrexate.

**Table 2:** Top 10 exosomal microRNAs differentially expressed between late-onset acute GVHD and non-GVHD.

Exosomal miRNAs	Fold Change	*p* Value
hsa-miR-423-5p	49.07	0.000198
hsa-miR-19a	37.33	0.000013
hsa-miR-142-3p	28.35	0.000198
hsa-miR-128	24.79	0.000111
hsa-miR-193b	16.12	0.000209
hsa-miR-30c	14.33	0.003157
hsa-miR-193a	12.56	0.000111
hsa-miR-191	12.3	0.000081
hsa-miR-125b	10.7	0.001064
hsa-miR-574-3p	6.13	0.000198

**Table 3:** Predicted target genes for miR-128 identified by miRTarBase.

miRNA	Experimentally Validated Targets by Reporter Assay (miRTarBase)
hsa-miR-128	BMI1, FBXW7, DCX, RELN, WEE1, TGFBR1, NEK2, SREBF1, SREBF2, ABCA1, ABCG1, RXRA
